# Supplementary material for: The Impact of Mixing Techniques on PMMA Bone Cement Subjected to Two Different Cooling Techniques: A Pilot Study of Thermal Management Strategies in Orthopedic Applications
Source: Biomedicines. 2025 Dec 12;13(12):3071. doi: 10.3390/biomedicines13123071 (PMC12730438; doi:10.3390/biomedicines13123071)
Supplement: Supplementary file 1 [file biomedicines-13-03071-s001.zip › biomedicines-3978128-supplementary.pdf]

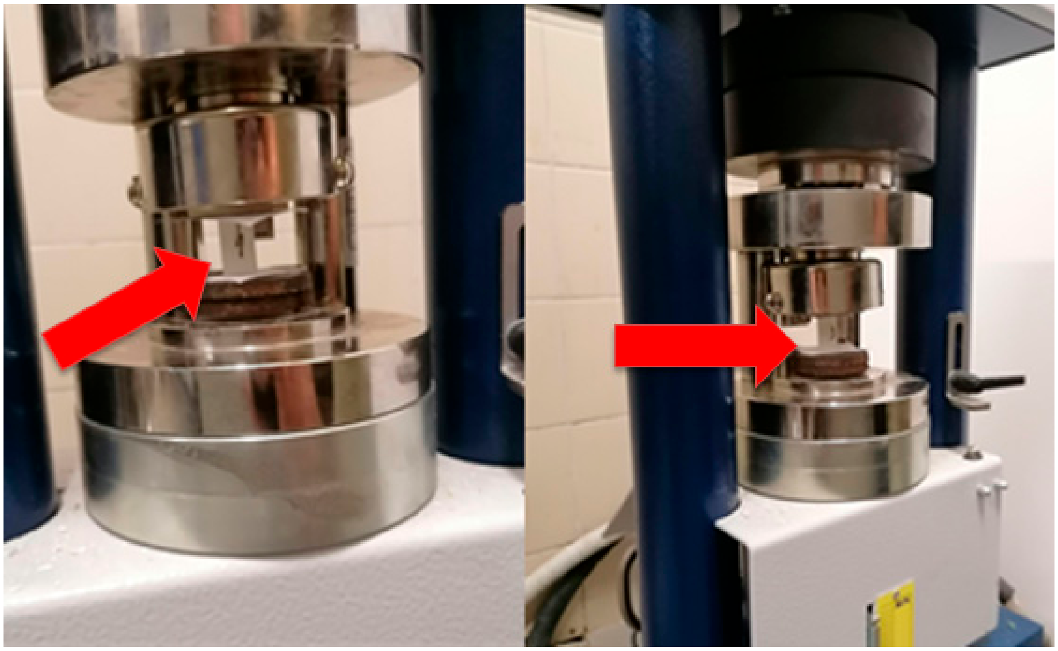

Figure S1. The MATEST exural frame during the cement testing with a cement cylinder inside. To serve as a base for the sample in order to be able to perform the experiment, two 6cm wide and 5 mm tall steel disks were stacked on top of one another.

Table S1. The average temperature values of each batch of cement cylinders at different moments in time during curing (HM - Hand mixed; VM - vacuum mixed)

Sample Group	5 min (°C)	SD	8 min (°C)	SD	10 min (°C)	SD	15 min (°C)	SD	18 min (°C)	SD
HM control	22.2	0.84	44.2	0.84	66.8	0.84	76.2	0.84	68.2	0.84
HM pre-cooled	5.2	0.84	30.6	0.89	53.2	0.84	63.6	0.89	57.2	0.80
HM saline irrigation	17.2	0.84	39	1.00	57	1.00	66	1.00	62.2	0.82
VM control	22.2	0.84	42.2	0.84	63.8	0.84	71.8	0.84	65.8	0.86
VM pre-cooled	5.2	0.84	28	0.71	49.8	0.84	58.8	0.84	54.8	0.84
VM saline irrigation	17.2	0.84	36.8	0.84	56.2	0.84	63.6	1.14	59.8	0.85

Table S2. Descriptive statistical analysis of the **c**ompressive strength of the three batches of PMMA bone cement (HM - Hand mixed; VM - vacuum mixed)

Sample Group	Mean (Mpa)	Std Dev (Mpa)	Min (Mpa)	Max (Mpa)	95% Confidence Interval
HM control	17.015	0.748	15.470	18.088	[16.665, 17.365]
HM pre-cooled	16.487	0.902	15.314	17.707	[16.065, 16.909]
HM saline irrigation	17.070	0.770	15.608	18.500	[16.710, 17.430]
VM control	79.726	1.536	76.939	82.527	[79.007, 80.445]

Sample Group	Mean (Mpa)	Std Dev (Mpa)	Min (Mpa)	Max (Mpa)	95% Confidence Interval
VM pre-cooled	77.021	1.989	74.382	80.071	[76.090, 77.952]
VM saline irrigation	78.485	1.440	75.841	81.080	[77.780, 79.128]
